# Comparison of the Duhamel Procedure and Transanal Endorectal Pull-through Procedure in the Treatment of Children with Hirschsprung’s Disease: A Systematic Review

**DOI:** 10.3390/jcm12206632

**Published:** 2023-10-20

**Authors:** Qi Wang, Yuanyuan Liang, Mengqi Luo, Liwei Feng, Bo Xiang

**Affiliations:** 1 Department of Pediatric Surgery and Laboratory of Pediatric Surgery, Rare Diseases Center, West China Hospital, Sichuan University, Chengdu 610041, China; qiwang_1991@wchscu.cn (Q.W.); liangyuanyuan@wchscu.cn (Y.L.); flw-yutao@163.com (L.F.); 2 State Key Laboratory of Oral Diseases, National Center for Stomatology, National Clinical Research Center for Oral Diseases, West China Hospital of Stomatology, Sichuan University, Chengdu 610041, China; luomengqi@stu.scu.edu.cn

**Keywords:** Hirschsprung’s disease, Duhamel, transanal endorectal pull-through, systematic review

## Abstract

**Objective:** To compare the Duhamel and transanal endorectal pull-through (TERPT) procedures in the treatment of children with Hirschsprung’s disease. Methods: Studies comparing the Duhamel and TERPT procedures were included until 22 July 2023. R software (version 4.3.0) was used to perform the meta-analysis. Results: Ten studies with a sum of 496 patients were included. The length of postoperative hospital stay and incidence of postoperative constipation were longer and higher after the Duhamel procedure than the TERPT procedure (*p* < 0.0001 and *p* = 0.0041, respectively). The incidence of postoperative anastomotic stricture was higher after the TERPT procedure than the Duhamel procedure (*p* = 0.0015). No significant differences were found in the incidence of postoperative fecal continence, fecal incontinence/soiling, anastomotic leak, or ileus between these two procedures. The operation time seemed to be similar for both procedures, but it became longer for the Duhamel procedure than the TERPT procedure after sensitivity analysis. While the incidence of postoperative enterocolitis seemed to be higher after the TERPT procedure, it became similar for both procedures in the subgroup analysis. Conclusions: The Duhamel procedure seems to be associated with a longer length of postoperative hospital stay, a higher incidence of postoperative constipation, and a lower incidence of postoperative anastomotic stricture than the TERPT procedure. However, the effect of these two procedures on the operation time and the incidence of postoperative enterocolitis remains unclear.

## 1. Introduction

With an incidence of 1 in 5000 live births, Hirschsprung’s disease (HSCR) is the most common gastrointestinal malformation causing intestinal obstruction in children [1]. To date, several surgical procedures have been proposed for the treatment of HSCR, including the Duhamel, Swenson, Rehbein, Soave, and transanal endorectal pull-through (TERPT) procedures [2,3,4,5,6]. The principle behind all of these procedures is the resection of the aganglionic bowel segment, bringing the ganglionic bowel to the anus, and preserving the function of the anal sphincter [7,8,9]. Among all procedures, the Duhamel procedure and the TERPT procedure are commonly used [10,11,12].

The Duhamel procedure was first proposed in 1956 by Bernard Duhamel; it involves a longitudinal, side-to-side anastomosis of the posterior wall of the native aganglionic rectum with the anterior wall of the recruited ganglionic proximal colon and an end-to-side anastomosis of the posterior wall of the ganglionic colon with the retained rectal end [2]. Then, Ikeda [4] and Soper [13] modified this procedure by using mechanical stapling devices for side-to-side colorectal anastomosis. Currently, the use of an Endo-Cutting Stapler for side-to-side anastomosis has made this procedure more convenient [14]. However, the residual aganglionic rectum and the Duhamel pouch in this procedure were reported to be related to postoperative complications such as constipation [15,16,17,18,19,20,21,22,23,24,25].

In 1964, Soave [5] described a new pull-through procedure for the treatment of HSCR; this involves the removal of aganglionic rectal mucosae with an end-to-end anastomosis of the recruited ganglionic proximal colon to the distal rectum just above the dentate line and through the retaining aganglionic rectal muscular cuff [5,6]. Then, a modified transanal one-stage Soave procedure was proposed in 1998 and was defined as the TERPT procedure [3]. Later, the TERPT procedure was also modified to a transanal Swenson-like procedure, requiring a full-thickness resection of the aganglionic distal colon and rectum just above the dentate line instead of the submucosal dissection [12,26,27,28]. Subsequently, several studies have reported favorable results of TERPT compared with other types of surgical procedures [12,29]. However, anal sphincter stretching and transanal mobilization of the rectum during this procedure were reported to increase the risk of complications such as soiling, constipation, and fecal incontinence [15,16,17,18,19,20,21,22,23,24,25].

In recent decades, the implementation of laparoscopy during the Duhamel and TERPT procedures has been reported to reduce trauma, loss of blood, intraoperative contamination, and intestinal adhesion [12,30]. However, there is still an ongoing debate about which procedure is preferable in the treatment of HSCR, and it is also unclear which procedure results in better outcomes. Therefore, we performed this systematic review and meta-analysis to compare the benefits and outcomes of the Duhamel and TERPT procedures in treating HSCR in children.

## 2. Methods

### 2.1. Search Strategy

Studies were identified by searching databases, including PubMed (Medline), EMBASE, Cochrane Library, Web of Science, and EBSCO Host until 22 July 2023. Searches were conducted using Medical Subject Headings (MeSH) and free text words, including (‘Hirschsprung’s disease’ OR ‘Hirschsprung disease’ OR ‘aganglionic megacolon’ OR ‘congenital megacolon’ OR ‘aganglionosis’) AND (‘Duhamel’ OR ‘surgery’ OR ‘pull through’ OR ‘transanal’). All articles were restricted to human studies written in English, and reference lists were searched for additional relevant articles. This systematic review was registered in PROSPERO with the registration number CRD42022357059.

### 2.2. Inclusion/Exclusion Criteria

Inclusion criteria for the studies were set as follows: (1) studies comparing the functional outcomes after the Duhamel procedure or TERPT procedure, including randomized controlled studies (RCTs), retrospective studies, prospective studies, and observational studies; (2) a detailed description of the patients’ information, outcomes, and complications. The exclusion criteria were set as follows: (1) duplicate publications; (2) studies reporting findings on the same group of patients; (3) studies reporting fewer than 10 patients in any group; (4) studies only regarding total colonic aganglionosis or adults; (5) studies reporting reoperation patients; (6) single-arm studies; (7) studies without detailed raw data (i.e., abstracts, letters, posters, case reports, conference reports, comments, reviews, and meta-analyses).

### 2.3. Data Extraction

The assessment of eligibility and risk of bias in included studies, as well as the raw data extraction, were performed by three independent reviewers (WQ, LYY, and LMQ). Discussion with other investigators (XB and FLW) resolved disagreements. The following data were collected for each suitable study: (1) general information: the first author, publication year, study location, study design, surgical technique, number of participants for each surgical procedure, participants’ gender and age; (2) baseline, benefits, and outcomes data: operation time, length of postoperative hospital stay, rate of postoperative fecal continence, fecal incontinence/soiling, constipation, enterocolitis, anastomotic stricture, leak, and postoperative ileus. Missing data were obtained by contacting the first and corresponding authors if possible.

### 2.4. Quality Assessment

Two independent authors (LYY and LMQ) assessed the quality of the included studies using the Newcastle—Ottawa Scale (NOS) [31].

### 2.5. Data Synthesis and Statistical Analysis

The protocol of this study was based on the Preferred Reporting Items for Systematic Reviews and Meta-Analyses (PRISMA) guidelines [32]. The meta-analysis was carried out using R software (version 4.3.0). For continuous data, the mean and standard deviation (SD) were extracted from the included articles. When the mean and SD were not directly reported, they were estimated from the sample size, median, and range according to the methods described in the Cochrane Handbook [33,34,35,36]. The effect sizes were reported as the weighted mean difference (WMD). For dichotomous data, the pooled odds ratios (ORs) and 95% confidence intervals (Cis) were calculated. Heterogeneity was evaluated based on *I*^2^ statistics. A common effect model (also referred to as a fixed-effect model) was used if *I*^2^ ≤ 50%, and a random effect model was used if *I*^2^ > 50%. Sensitivity analysis was conducted using a leave-one-out approach. Statistical significance was determined by a probability value of < 0.05.

## 3. Results

### 3.1. Search Process and Characteristics of the Included Studies

**Figure 1:**
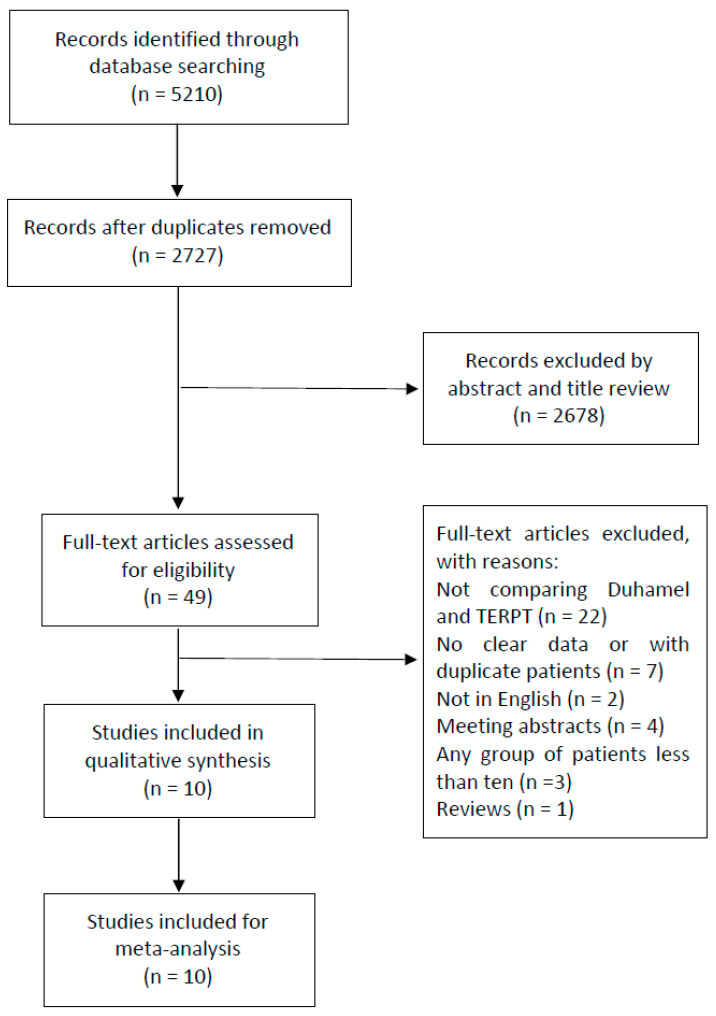
Flow chart of the study selection process.

The search and screening process in this study is presented in Figure 1. A total of 5210 studies were identified in the primary database search. Following the removal of duplicates and the review of titles and abstracts, only 49 studies were left to assess for eligibility. Finally, 10 articles met the inclusion criteria after careful assessment by a full-text review [15,16,17,18,19,20,21,22,23,24]. Moreover, because the complication data of patients under 18 years old were not available in the study by Davison et al. [25], this article was not included in the meta-analysis. Other articles were excluded for the reasons shown in Figure 1.

Finally, a total of 496 patients (*n* = 285 for the Duhamel procedure and *n* = 211 for the TERPT procedure) were included in the meta-analysis. Of these, 133 patients were from prospective studies [15,18,24], and the remaining 363 patients were from retrospective studies [16,17,19,20,21,22,23]. Furthermore, in the included articles reporting the extent of disease, 40 patients had short-segment HSCR, 230 patients had rectosigmoid HSCR, and 23 patients had long-segment HSCR [15,17,19,20,21]. Other characteristics, including quality assessments of the included studies, are summarized in Table 1.

The search and screening process of eligible studies, and the number of studies at each stage.

### 3.2. Operation Time and Length of Postoperative Hospital Stay

**Figure 2:**
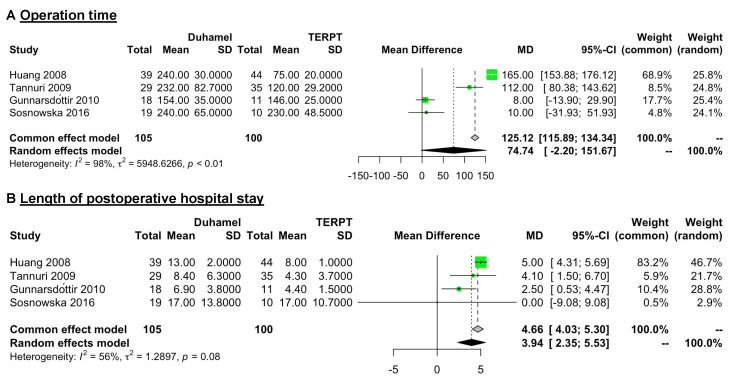
Forest plot comparing operation time (minute) and length of postoperative hospital stay (day) between patients treated with the Duhamel procedure or the TERPT procedure. The overall pooled analysis revealed that (**A**) the operation time was similar between these two surgical procedures (WMD = 74.74 min, 95% CI = −2.20 to 151.67, *p* = 0.0569), and (**B**) the length of postoperative hospital stay was longer in patients treated with the Duhamel procedure than in those treated with the TERPT procedure (WMD = 3.94 days, 95% CI = 2.35 to 5.53, *p* < 0.0001) [17,19,20,22].

Four studies (105 patients for the Duhamel procedure and 100 patients for the TERPT procedure) reported detailed data on the operation time and length of postoperative hospital stay [17,19,20,22] (Table 2). A random effect model of analysis was used for a high level of heterogeneity in these data (*I*^2^ = 98%, *p* < 0.01 in operation time data and *I*^2^ = 56%, *p* = 0.08 in length of postoperative hospital stay data). The overall pooled analysis revealed that the operation time was similar between these two surgical procedures (WMD = 74.74 min, 95% CI = −2.20 to 151.67, *p* = 0.0569) (Figure 2A), while the length of postoperative hospital stay was longer in patients treated with the Duhamel procedure than in those treated with the TERPT procedure (WMD = 3.94 days, 95% CI = 2.35 to 5.53, *p* < 0.0001) (Figure 2B).

However, during the sensitivity analysis, we found that although the *I^2^* did not change significantly (range: 93.3–98.9%), after omitting the data of operation time from Gunnarsdóttir et al. [20] and Sosnowska et al. [22] (which comprised fewer than 20 patients in each group), the meta-analysis results indicated that the operation time was longer in patients treated with the Duhamel procedure than in those treated with the TERPT procedure (WMD = 97.44 min, 95% CI = 8.86 to 186.03, *p* = 0.0311 and WMD = 95.22 min, 95% CI = 4.20 to 186.25, *p* = 0.0403, respectively). The detailed results are presented in the Supplementary Materials (Figure S1A–C).

### 3.3. Postoperative Fecal Continence, Fecal Incontinence/Soiling, and Constipation

**Figure 3:**
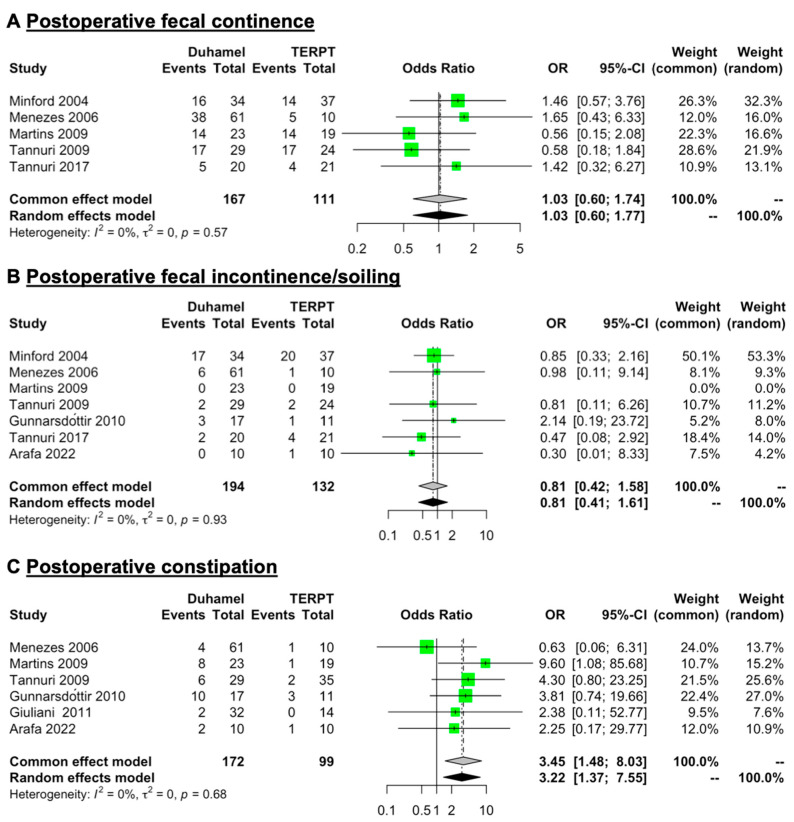
Forest plot comparing the rate of postoperative fecal continence, fecal incontinence/soiling, and constipation between patients treated with the Duhamel procedure or the TERPT procedure. The overall pooled analysis revealed that (**A**,**B**) the rates of postoperative fecal continence and fecal incontinence/soiling were similar between the two surgical procedures (OR = 1.03, 95% CI = 0.60 to 1.74, *p* = 0.9218 and OR = 0.81, 95% CI = 0.42 to 1.58, *p* = 0.5447, respectively), (**C**) while the rate of postoperative constipation was higher in patients treated with the Duhamel procedure than in those treated with the TERPT procedure (OR = 3.45, 95% CI = 1.48 to 8.03, *p* = 0.0041) [15,16,18,19,20,21,23,24].

Five studies (167 patients for the Duhamel procedure and 111 patients for the TERPT procedure) reported detailed data on postoperative fecal continence [15,16,18,19,23] (Table 2). The mean rate of postoperative fecal continence was 53.89% (range: 25–62.30%) for the Duhamel procedure and 50.03% (range: 19.05–73.68%) for the TERPT procedure. Seven studies (194 patients for the Duhamel procedure and 132 patients for the TERPT procedure) reported detailed data of postoperative fecal incontinence/soiling [15,16,18,19,20,23,24] (Table 2). The mean rate of postoperative fecal incontinence/soiling was 15.46% (range: 0–50%) for the Duhamel procedure and 21.97% (range: 0–54.05%) for the TERPT procedure. A common (fixed) effect model of analysis was used for a low level of heterogeneity in these data (*I*^2^ = 0%, *p* = 0.57 in postoperative fecal continence data and *I*^2^ = 0%, *p* = 0.93 in postoperative fecal incontinence/soiling data). The overall pooled analysis revealed that the rates of postoperative fecal continence and fecal incontinence/soiling were similar between these two surgical procedures (OR = 1.03, 95% CI = 0.60 to 1.74, *p* = 0.9218 and OR = 0.81, 95% CI = 0.42 to 1.58, *p* = 0.5447, respectively) (Figure 3A,B).

Six studies (172 patients for the Duhamel procedure and 99 patients for the TERPT procedure) reported detailed data on postoperative constipation [16,18,19,20,21,24] (Table 2). The mean rate of postoperative constipation was 18.60% (range: 6.25–58.82%) for the Duhamel procedure and 8.08% (range: 0–27.27%) for the TERPT procedure. A common (fixed) effect model of analysis was used for a low level of heterogeneity in these data (*I^2^* = 0%, *p* = 0.68). The overall pooled analysis revealed that the rate of postoperative constipation was higher in patients treated with the Duhamel procedure than in those treated with the TERPT procedure (OR = 3.45, 95% CI = 1.48 to 8.03, *p* = 0.0041) (Figure 3C).

### 3.4. Postoperative Enterocolitis

**Figure 4:**
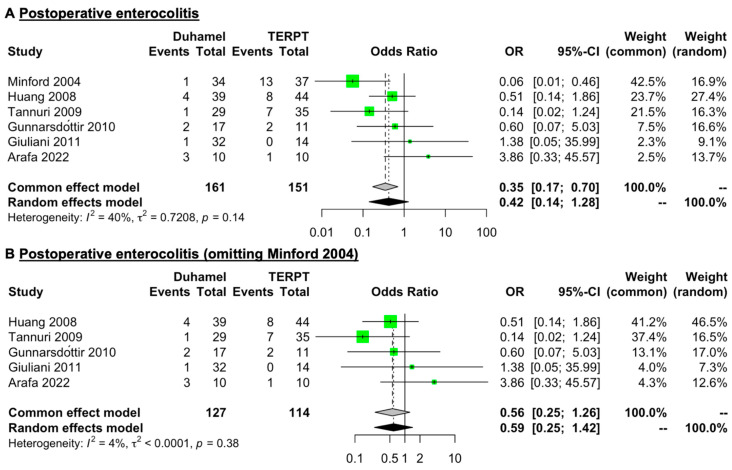
Forest plot comparing the rate of postoperative enterocolitis between patients treated with the Duhamel procedure or the TERPT procedure. (**A**) The overall pooled analysis revealed that the rate of postoperative enterocolitis was lower in patients treated with the Duhamel procedure than in those treated with the TERPT procedure (OR = 0.35, 95% CI = 0.17 to 0.70, *p* = 0.0033). (**B**) After omitting the data from Minford, the *I*^2^ index decreased to 4% (*p* = 0.38); the meta-analysis results indicated that the rate of postoperative enterocolitis was similar between the two surgical procedures (OR = 0.56, 95% CI = 0.25 to 1.26, *p* = 0.1611) [15,17,19,20,21,24].

Six studies (161 patients for the Duhamel procedure and 151 patients for the TERPT procedure) reported detailed data on postoperative enterocolitis [15,17,19,20,21,24] (Table 2). The mean rate of postoperative enterocolitis was 7.45% (range: 2.94–30%) for the Duhamel procedure and 20.53% (range: 0–35.14%) for the TERPT procedure. A common (fixed) effect model of analysis was used for a low level of heterogeneity in these data (*I*^2^ = 40%, *p* = 0.14). The overall pooled analysis revealed that the rate of postoperative enterocolitis was lower in patients treated with the Duhamel procedure than in those treated with the TERPT procedure (OR = 0.35, 95% CI = 0.17 to 0.70, *p* = 0.0033) (Figure 4A).

During the sensitivity analysis, we found that the data of Minford et al. [15] were the main source of heterogeneity. After omitting the data of postoperative enterocolitis from Minford et al. [15], the *I*^2^ index decreased to 4% (*p* = 0.38), and the meta-analysis results indicated that the rate of postoperative enterocolitis was similar between these two surgical procedures (OR = 0.56, 95% CI = 0.25 to 1.26, *p* = 0.1611) (Figure 4B). The detailed results of the sensitivity analysis of postoperative enterocolitis are presented in the Supplementary Materials (Figure S2).

### 3.5. Postoperative Anastomotic Stricture, Anastomotic Leak, and Ileus

**Figure 5:**
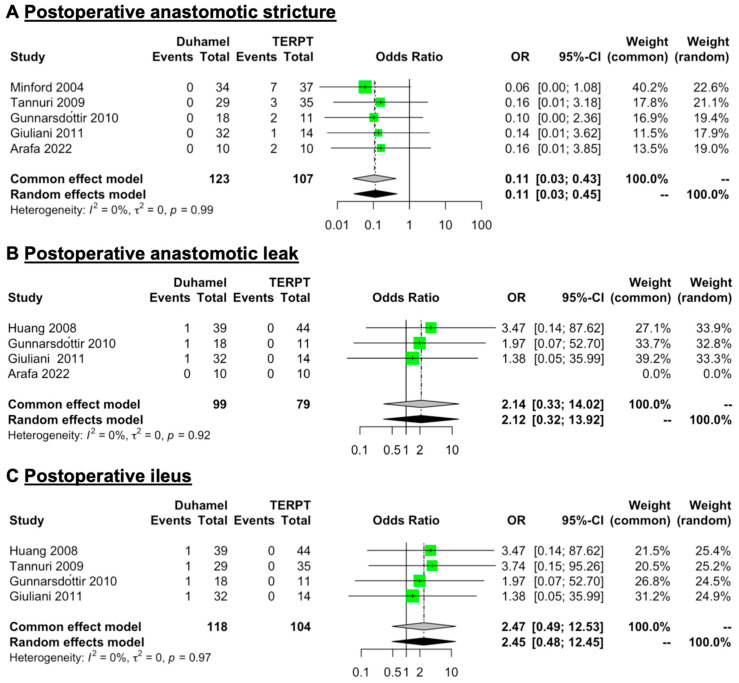
Forest plot comparing the rate of postoperative anastomotic stricture, anastomotic leak, and ileus between patients treated with the Duhamel procedure or the TERPT procedure. The overall pooled analysis revealed that (**A**) the rate of postoperative anastomotic stricture was lower in patients treated with the Duhamel procedure than in those treated with the TERPT procedure (OR = 0.11, 95% CI = 0.03 to 0.43, *p* = 0.0015), (**B**,**C**) while the rates of postoperative anastomotic leak and ileus were similar between the two surgical procedures (OR = 2.14, 95% CI = 0.33 to 14.02, *p* = 0.4257 and OR = 2.47, 95% CI = 0.49 to 12.53, *p* = 0.2747, respectively) [15,17,19,21,24].

Five studies (123 patients for the Duhamel procedure and 107 patients for the TERPT procedure) reported detailed data on postoperative anastomotic stricture [15,19,20,21,24] (Table 2). The mean rate of postoperative anastomotic stricture was 0% for the Duhamel procedure and 14.02% (range: 7.14–20%) for the TERPT procedure. A common (fixed) effect model of analysis was used for a low level of heterogeneity in these data (*I*^2^ = 0%, *p* = 0.99). The overall pooled analysis revealed that the rate of postoperative anastomotic stricture was lower in patients treated with the Duhamel procedure than in those treated with the TERPT procedure (OR = 0.11, 95% CI = 0.03 to 0.43, *p* = 0.0015) (Figure 5A).

Four studies (99 patients for the Duhamel procedure and 79 patients for the TERPT procedure) reported detailed data on postoperative anastomotic leak [17,20,21,24], while four studies (118 patients for the Duhamel procedure and 104 patients for the TERPT procedure) reported detailed data on postoperative ileus [17,19,20,21] (Table 2). The mean rates of postoperative anastomotic leak and ileus were 3.03% (range: 0–5.56%) and 3.39% (range: 2.56–5.56%) for the Duhamel procedure, respectively. The relevant rates were both 0% for the TERPT procedure. A common (fixed) effect model of analysis was used for a low level of heterogeneity in these data (*I*^2^ = 0%, *p* = 0.92 in postoperative anastomotic leak data and *I*^2^ = 0%, *p* = 0.97 in postoperative ileus data). The overall pooled analysis revealed that the rates of postoperative anastomotic leak and ileus were similar between the two surgical procedures (OR = 2.14, 95% CI = 0.33 to 14.02, *p* = 0.4257 and OR = 2.47, 95% CI = 0.49 to 12.53, *p* = 0.2747, respectively) (Figure 5B,C).

### 3.6. Sensitivity Analysis

A sensitivity analysis of the operation time and postoperative enterocolitis has been described above. In other sensitivity analyses, excluding each study in turn did not affect the meta-analysis results for the length of postoperative hospital stay or the rates of postoperative fecal continence, fecal incontinence/soiling, anastomotic stricture, constipation, anastomotic leak, and ileus.

## 4. Discussion

The Duhamel procedure and the TERPT procedure are commonly used for the treatment of HSCR [10,11,12]. The advantages of the Duhamel procedure are good visibility throughout the entire process and limited anal stretching, while the advantages of the TERPT procedure are minimal invasion and good cosmesis [12]. Through decades of use, multiple studies have reported inconsistent results on the benefits and outcomes of these two procedures, and no consensus has been reached about which procedure is significantly better in terms of general and disease-specific outcomes [10,11,12,15,16,17,18,19,20,21,22,23,24]. Therefore, we conducted a systematic review and meta-analysis that was as comprehensive as possible to evaluate the benefits and outcomes of the Duhamel and TERPT procedures in treating children with HSCR.

Initially, the results of the meta-analysis indicated that the operation time between the Duhamel and TERPT procedures was similar, which was consistent with prior systematic reviews [10,37]. However, we found that the raw data of operation time from four included studies [17,19,20,22] seemed to indicate that the operation time was longer in the Duhamel procedure. Considering that the heterogeneity between these four studies was high (*I*^2^ = 98%, *p* < 0.01), we performed a sensitivity analysis and found that although the *I^2^* did not change significantly, the meta-analysis results indicated that the operation time was longer for the Duhamel procedure than for the TERPT procedure after omitting the data from Gunnarsdóttir et al. [20] and Sosnowska et al. [22]. Notably, the number of patients in each group reported by Gunnarsdóttir et al. [20] and Sosnowska et al. [22] was fewer than 20 cases, which was less than the required number of cases recommended by an expert workshop to reach the learning curve plateau for the Duhamel procedure [12]. Therefore, we believe this may be a reason for the similar operation times between the two procedures in these two studies [20,22] and the uncertain results of the meta-analysis. Another reason for this result might be that the operation time of the TERPT procedure in the Gunnarsdóttir et al. [20] study included a waiting time of approximately 45 min for the frozen section analysis, which was not required for the Duhamel procedure.

Although the result of the comparison of the operation times was uncertain, the length of postoperative hospital stay seemed to be longer in patients treated with the Duhamel procedure, which was also consistent with previous studies [10,37,38]. In addition, Gunnarsdóttir et al. [20] reported that patients treated with the TERPT procedure started oral feeding and had bowel movements sooner than those in the Duhamel group. Hence, the results may suggest that patients treated with the TERPT procedure could recover faster, but further studies are needed to support this point.

As with several previous studies [11,16,18,19,20,21,24,37,38], the results of the present study also demonstrated that the rate of postoperative constipation seems to be higher in patients treated with the Duhamel procedure. The residue of dysfunctional aganglionic intestinal tissue was reported to be closely related to the occurrence of postoperative constipation [12]. In particular, the Duhamel procedure retained part of the aganglionic rectal segment for anastomosis with the ganglionic colon; in the TERPT procedure, an end-to-end anastomosis was made just above the dentate line, while almost all aganglionic intestinal tissues were removed [2,3,6,8,12]. In addition, the Duhamel procedure was favorable when treating children with long-segment HSCR, which may also contribute to a higher rate of postoperative complications [12]. Although the rate of postoperative constipation was relatively lower, a short rectal muscular cuff—no longer than 5 cm—was recommended in the TERPT procedure to avoid this complication [12]. Even so, several studies have also reported that defecation patterns gradually improve with age in children with HSCR after the pull-through procedure [39,40].

Another reported postoperative complication that was similar to constipation was anastomotic stricture, which seemed to be higher in patients treated with the TERPT procedure. This may be related to the annular dissection and anastomosis in the anorectum of this procedure; other reported risk factors were anastomotic ischemia, anastomotic leakage, and cuff ischemia [7,39,40,41]. Older children were also reported to be more likely to experience this complication than younger children [11,20,42]. However, regular anal dilation with Hegar dilators has been reported to reduce the occurrence of anastomotic stricture and improve symptoms [11,24,43]. Therefore, prophylactic anal bouginage with Hegar dilators was recommended at 2 weeks after the TERPT procedure [43].

One of the most serious postoperative complications was enterocolitis. The etiology of enterocolitis is unknown and is probably multifactorial [7]. Several risk factors have been identified for enterocolitis, including younger age, longer segment disease, trisomy 21, anastomotic stricture, and malnutrition [7,12,44]. Currently, the reported incidence of enterocolitis after the Duhamel procedure or the TERPT procedure is controversial in published studies [15,17,19,20,21,24]. Some studies have reported that the incidence of enterocolitis was higher after the TERPT procedure [10,15,17,19,20], while other studies have reported contrary results [21,24,45]. Initially, our meta-analysis showed that the rate of enterocolitis was higher in patients treated with the TERPT procedure, but after omitting the most heterogeneous article, the analysis showed that the rate of postoperative enterocolitis was similar between the two surgical procedures. Significant differences in age at operation, length of follow-up, and extent of disease among the included studies may have contributed to this result.

This study has various limitations. First, most of the included studies were retrospectively designed with small sample sizes and different extent of disease, which could add bias to this meta-analysis. Second, the included studies spanned a wide timeframe (2004–2022). In the intervening years, the improvement of surgical techniques, perioperative management, complication prevention, and treatment would inevitably affect the overall prognosis of patients. This can also lead to bias and affect the analysis results. Third, the gap in age at operation among the included patients was also large (0.3–180 months), which may influence the incidence of complications (such as constipation and enterocolitis) and create bias. Fourth, variability in surgical techniques in the included studies, such as length of the rectal cuff, Swenson or Soave model for transanal procedure, and mechanical or manual coloanal anastomosis, may also create bias. Finally, the definition and evaluation criteria of complications were not standardized (especially for fecal continence, fecal incontinence, constipation, and enterocolitis), which may also introduce bias and impact the results of the analysis.

## 5. Conclusions

In conclusion, based on the data reported, the findings of this study indicated that the Duhamel procedure seems to be associated with a longer length of postoperative hospital stay and a higher incidence of postoperative constipation. The TERPT procedure seems to be associated with a higher incidence of postoperative anastomotic stricture. For the incidence of postoperative fecal continence, fecal incontinence/soiling, anastomotic leak, and ileus, these two procedures seem to be similar. However, based on the data analyzed, the differences in operation time and rate of postoperative enterocolitis between these two procedures were unclear. Further prospective studies with a larger sample size and adequate follow-up are needed to obtain more definitive conclusions.

## Figures and Tables

**Table 1:** Characteristics of the included studies.

First Author, Publication Year, Country	Journal	Study Design	Surgical Technique (*n*)	Age at Operation (Month)	Male:Female (*n*)	Extent of Disease (*n*)			Length of FolLow Up (Month)	Main Outcome and Complication Measurements	NOS Score
						Short	RS	Long			
Minford [15], (2004), UK	J Pediatr Surg	Prospective	Duhamel (*n* = 34)	84 (median) (age at scoring)	25:9	0	24	10	NA	Morbidity and mortality, enterocolitis, stricture, rectal spur, myectomy, late stoma formation and operative failure, functional outcome score.	7
			TERPT (*n* = 37)	72 (median) (age at scoring)	27:10	0	27	10	NA		
Menezes [16], (2006), Ireland	Pediatr Surg Int	Retrospective	Duhamel (*n* = 61)	NA	NA	NA	NA	NA	NA	Long-term bowel function, soiling, constipation.	4
			TERPT (*n* = 10)	NA	NA	NA	NA	NA	NA		
Huang [17], (2008), China	J Pediatr Surg	Retrospective	Duhamel (*n* = 39)	27.6 (mean) (range: 3 to 120)	34:5	21	18	0	12 to 60	Perioperative therapeutic effect, rating of bowel movements, anastomotic leak, incision infection, adhesive ileus, enterocolitis, death, anorectal manometry.	6
			TERPT (*n* = 44)	NA	NA	14	30	0	12 to 60		
Martins [18], (2009), Brazil	Acta Cir Bras	Prospective	Duhamel (*n* = 23)	104.4 (mean) (range: 24 to 180)	NA	NA	NA	NA	NA	Constipation, continence, anorectal manometry	5
			TERPT (*n* = 19)	60 (mean) (range: 12 to 108)	NA	NA	NA	NA	NA		
Tannuri [19], (2009), Brazil	J Pediatr Surg	Retrospective	Duhamel (*n* = 29)	42 (mean) (range: 6 to 110)	NA	0	29	0	2 to 168	Operating time, post operative hospital stay, enterocolitis, wound infection, mortality, stooling patterns, postoperative continence, perineal dermatitis.	5
			TERPT (*n* = 35)	11 (mean) (range: 0.3 to 72)	NA	0	35	0	2 to 72		
Gunnarsdóttir [20], (2010), Sweden	Eur J Pediatr Surg	Retrospective	Duhamel (*n* = 18)	5.6 (mean) (range: 1 to 23)	15:3	0	18	0	25 to 45	Operative time, perioperative bleeding, time of oral feeding and bowel movement postoperatively, the length of hospital stay, enterocolitis.	7
			TERPT (*n* = 11)	4.8 (mean) (range: 1 to 24)	7:4	0	11	0	25 to 48		
Giuliani [21], (2011), Italy	J Laparoendosc Adv S	Retrospective	Duhamel (*n* = 32)	14.61 (mean)	9:1	3	28	1	≥12	Operative time, length of hospital stay, postoperative start of oral feeding, postoperative enterocolitis, incidence of severe constipation or incontinence.	6
			TERPT (*n* = 14)	4.67 (mean)	8:1	2	10	2	26 (mean)		
Sosnowska [22], (2016), Poland	Prz Gastroenterol	Retrospective	Duhamel (*n* = 19)	49 (mean)	NA	NA	NA	NA	NA	Operative time of radical surgery, length of hospitalisation after radical surgeryl, number and cause of complications.	6
			TERPT (*n* = 10)	16 (mean)	NA	NA	NA	NA	NA		
Tannuri [23] , (2017), Brazil	J Pediatr Surg	Retrospective	Duhamel (*n* = 20)	41 (median) (range: 6 to 110)	3:1	NA	NA	NA	6 to 60	The Fecal Continence Index (FCI) questionnaire and the Assessment of Quality of Life in Children and Adolescents with Fecal Incontinence (AQLCAFI) questionnaire	4
			TERPT (*n* = 21)	10 (median) (range: 0.3 to 72)	16:5	NA	NA	NA	6 to 55		
Arafa [24], (2022), Egypt	Front Surg	Prospective	Duhamel (*n* = 10)	36 (mean)	NA	NA	NA	NA	12	Operative time, length of hospital stay, leakage, perianal excoriation, postoperative enterocolitis, constipation, anal stenosis, spur formation and fecal incontinence.	4
			TERPT (*n* = 10)	36 (mean)	NA	NA	NA	NA	12		

***n*:** Number of patients; ***TERPT***: transanal endorectal pull-through; ***RS:*** rectosigmoid; ***NA***: not available; ***NOS***: Newcastle–Ottawa Scale.

**Table 2:** Summary of main outcomes and complications.

First Author, Publication Year, Country	Journal	Surgical Technique (*n*)	Operation Time (Minute) ^a^	Length of Postoperative Hospital Stay (Day) ^a^	Fecal Continence (*n*)	Fecal Incontinence/ Soiling (*n*)	Constipation (*n*)	Postoperative Enterocolitis (*n*)	Anastomotic Stricture (*n*)	Anastomotic Leak (*n*)	Post Operative Ileus (*n*)
Minford [15], (2004), UK	J Pediatr Surg	Duhamel (*n* = 34)	NA	NA	16	17	NA	1	0	NA	NA
		TERPT (*n* = 37)	NA	NA	14	20	NA	13	7	NA	NA
Menezes [16], (2006), Ireland	Pediatr Surg Int	Duhamel (*n* = 61)	NA	NA	38	6	4	NA	NA	NA	NA
		TERPT (*n* = 10)	NA	NA	5	1	1	NA	NA	NA	NA
Huang [17], (2008), China	J Pediatr Surg	Duhamel (*n* = 39)	240 ± 30	13 ± 2	NA	NA	NA	4	NA	1	1
		TERPT (*n* = 44)	75 ± 20	8 ± 1	NA	NA	NA	8	NA	0	0
Martins [18], (2009), Brazil	Acta Cir Bras	Duhamel (*n* = 23)	NA	NA	14	0	8	NA	NA	NA	NA
		TERPT (*n* =19)	NA	NA	14	0	1	NA	NA	NA	NA
Tannuri [19], (2009), Brazil	J Pediatr Surg	Duhamel (*n* = 29)	232 ± 82.7	8.4 ± 6.3	17	2	6	1	0	NA	1
		TERPT (*n* =35)	120 ± 29.2	4.3 ± 3.69	17 (among 24 patients)	2 (among 24 patients)	2	7	3	NA	0
Gunnarsdóttir [20], (2010), Sweden	Eur J Pediatr Surg	Duhamel (*n* = 18)	154 ± 35	6.9 ± 3.8	NA	3 (among 17 patients)	10 (among 17 patients)	2 (among 17 patients)	0	1	1
		TERPT (*n* = 11)	146 ± 25	4.4 ± 1.5	NA	1	3	2	2	0	0
Giuliani [21], (2011), Italy	J Laparoendosc Adv S	Duhamel (*n* = 32)	257 (mean)	6.8 (mean)	NA	NA	2	1	0	1	1
		TERPT (*n* = 14)	195 (mean)	4.4 (mean)	NA	NA	0	0	1	0	0
Sosnowska [22], (2016), Poland	Prz Gastroenterol	Duhamel (*n* = 19)	240 (mean)	17 (mean)	NA	NA	NA	NA	NA	NA	NA
		TERPT (*n* = 10)	230 (mean)	17 (mean)	NA	NA	NA	NA	NA	NA	NA
Tannuri [23], (2017), Brazil	J Pediatr Surg	Duhamel (*n* = 20)	NA	NA	5	2	NA	NA	NA	NA	NA
		TERPT (*n* = 21)	NA	NA	4	4	NA	NA	NA	NA	NA
Arafa [24], (2022), Egypt	Front Surg	Duhamel (*n* = 10)	NA	NA	NA	0	2	3	0	0	NA
		TERPT (*n* = 10)	NA	NA	NA	1	1	1	2	0	NA

***n*:** Number of patients; ^a^: mean ± standard deviation; ***TERPT*:** transanal endorectal pull-through; ***NA***: not available.

## Data Availability

All data have been provided in the article.

## Supplementary Materials

The following supporting information can be downloaded at: https://www.mdpi.com/article/10.3390/jcm12206632/s1, Figure S1: Forest plot of sensitivity analysis of operation time data. After omitting the data from Gunnarsdóttir et al. or Sosnowska et al., the meta-analysis results would indicated that the operation time was longer in patients treated with the Duhamel procedure than in those treated with the TERPT procedure (WMD = 97.44 min, 95% CI = 8.86 to 186.03, *p* = 0.0311 and WMD = 95.22 min, 95% CI = 4.20 to 186.25, *p* = 0.0403, respectively).
