# Supplementary material for: Comparison of the Duhamel Procedure and Transanal Endorectal Pull-through Procedure in the Treatment of Children with Hirschsprung’s Disease: A Systematic Review
Source: J Clin Med. 2023 Oct 20;12(20):6632. doi: 10.3390/jcm12206632 (PMC10607700; doi:10.3390/jcm12206632)
Supplement: Supplementary file 1 [file jcm-12-06632-s001.zip › jcm-2591672-supplementary.pdf]

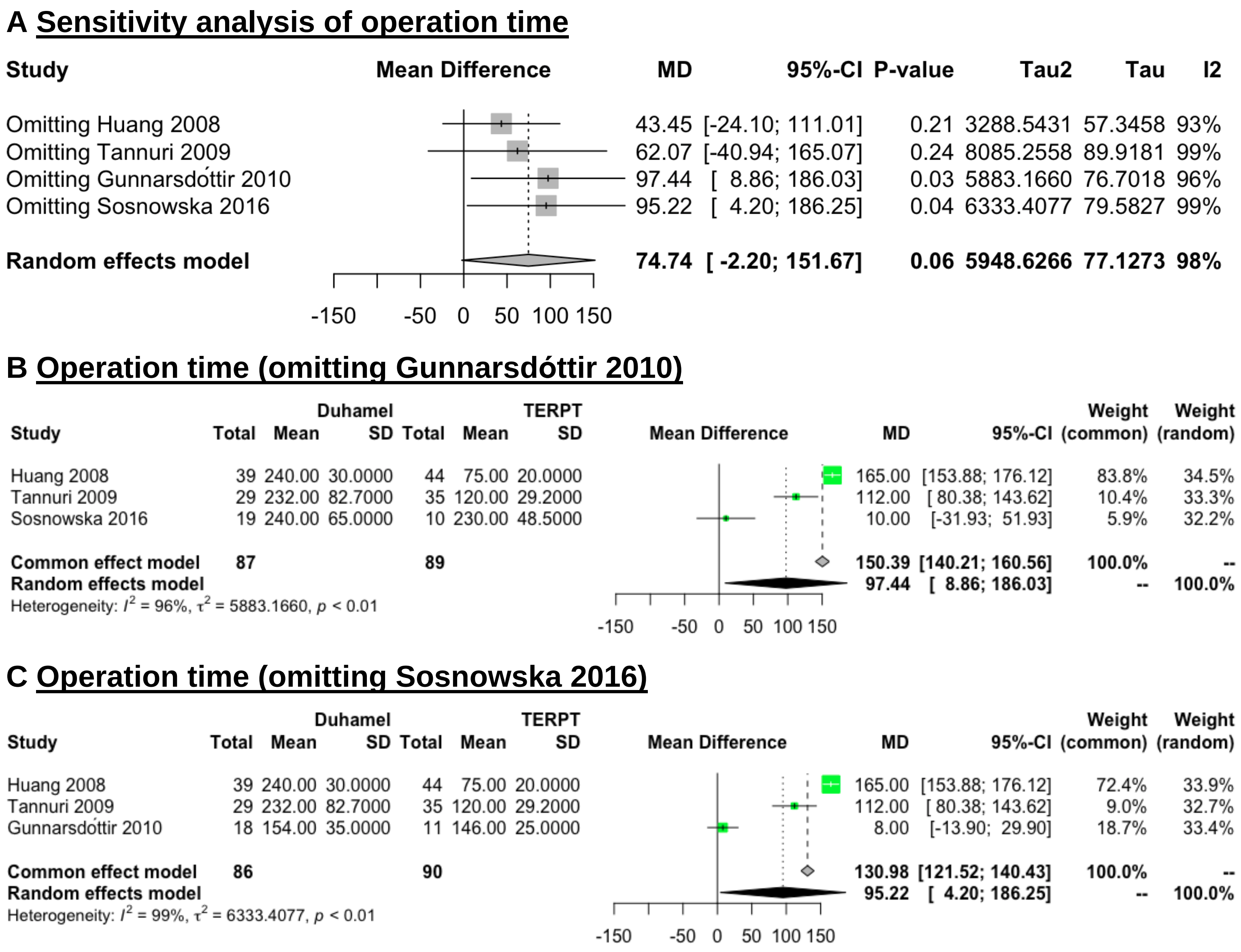

Figure S1 Forest plot of sensitivity analysis of operation time data. After omitting the data from Gunnarsdóttir et al. or Sosnowska et al., the meta-analysis results would indicated that the operation time was longer in patients treated with the Duhamel procedure than in those treated with the TERPT procedure (WMD = 97.44 min, 95% CI = 8.86 to 186.03, P = 0.0311 and WMD = 95.22 min, 95% CI = 4.20 to 186.25, P = 0.0403, respectively) [17,19,20,22].

Sensitivity analysis of postoperative enterocolitis

Study	OR	95%-CI	P-value	Tau2	Tau	I2
Omitting Minford 2004	0.56	[0.25; 1.26]	0.16	< 0.0001	0.0011	4%
Omitting Huang 2008	0.30	[0.13; 0.69]	< 0.01	1.5266	1.2356	51%
Omitting Tannuri 2009	0.40	[0.19; 0.86]	0.02	0.9656	0.9826	45%
Omitting Gunnarsdóttir 2010	0.33	[0.15; 0.69]	< 0.01	1.2867	1.1343	51%
Omitting Giuliani 2011	0.32	[0.16; 0.67]	< 0.01	0.9097	0.9538	48%
Omitting Arafa 2022	0.26	[0.12; 0.57]	< 0.01	0.1904	0.4364	17%
**Common effect model**	**0.35**	**[0.17; 0.70]**	**< 0.01**	**0.7208**	**0.8490**	**40%**

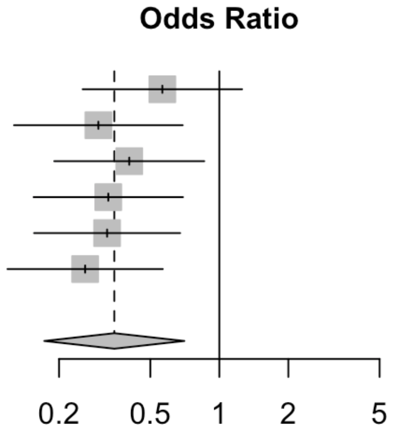

Figure S2 Forest plot of sensitivity analysis of postoperative enterocolitis data [15,17,19–21,24].
